# Enablers and barriers to implementing care quality improvement program in nursing homes in China

**DOI:** 10.1186/s12877-021-02488-0

**Published:** 2021-10-07

**Authors:** Yinan Zhao, Lulu Liao, Hui Feng, Huijing Chen, Hongting Ning

**Affiliations:** 1 Xiangya School of Nursing, Central South University, Changsha, Hunan China; 2 Xiangya-Oceanwide Health Management Research Institute, Central South University, Changsha, China; 3 National Clinical Research Center for Geriatric Disorders, Xiangya Hospital, Changsha, China

**Keywords:** Mentoring, Quality improvement, Nursing home, Qualitative research

## Abstract

**Objective:**

To explore the perspectives of key stakeholders on necessary factors to implement care quality improvement program.

**Methods:**

We conducted qualitative descriptive research in eight nursing homes in four major prefecture-level cities of Changsha, Xiangtan, Zhuzhou, and Yueyang. Data of 50 clinical nurses and 64 nurse assistants were included and analyzed. Ethical approval was given by the medical ethics committee of Chinese Clinical Trial Registry (No. ChiCTR-IOC-17013109, https://www.chictr.org.cn/index.aspx). One-to-one interviews were used with the nursing managers, and separate focus group discussions were used with the clinical nurses and nurse assistants. All of the interviews were audio recorded and later transcribed verbatim. In addition, the first author documented the responses of every participant in the field notes during the interviews and focus groups.

**Results:**

The participants’ perspectives were characterized by two main themes: (1) enablers, with four subthemes of “organizational support”, “the evidence-based practice ability”, “proactivity”, “nursing supervision and feedback;” and (2) barriers, with five sub-themes of “low educational background”, “the limitations of self-role orientation”, “resistance to change”, “lack of job motivation”, and “organizational constraints”.

**Conclusion:**

These findings recognize factors at the organizational level, staff level and societal level that are necessary to implement effective mentoring. The results of this study can provide reference for nursing home in improving nursing management quality, formulating, implementing and revising training policies.

## Background

The population of older people is increasing remarkably; the ageing population will increase by one million annually until the end of 2025 [1]. It has also been estimated that more than 100 million people over the age of 60 suffer from chronic diseases, which has become a major challenge for health systems [2, 3]. Due to heavy demand for nursing care, many older people have to change their conventional home-based care to professional nursing homes (NHs) [4]. Therefore, increasing attention has been paid to the quality of care in nursing homes.

Despite the availability of high-quality care has been a crucial area of concern in the development of nursing homes, [5] little practical guidance exists for nursing home staff to effect change [6]. However, due to the rising number of elderly people, older age, high disease rates and understaffing, increased complexity of care, lack of care skills, and the higher expectations regarding the quality of care, meanwhile, a lack of workforce development to achieve evidence-based quality of care and the improper attitudes and misconduct of nursing staff are also an important barrier to providing high-quality care [7–9]. NHs are usually perceived as resource-poor settings for quality improvement [10–13].

**Fig. 1:**
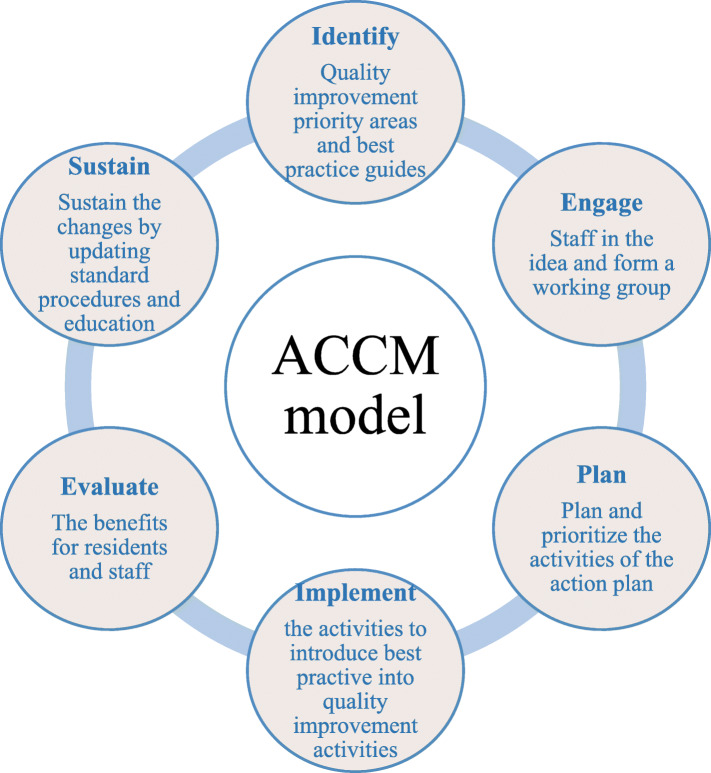
Elements of the ACCM intervention and their definition

Currently, using clinical mentors (CM) or champions as models of quality change has been widely reported in the evidence-based quality improvement literature, and this model has been recognized as a successful model in acute care Settings and geriatric care Settings worldwide [14, 15]. The Australian government initiated a large-scale evidence-based quality improvement project in aged care entitled ‘Clinical Mentoring: From Evidence Base to Outcomes for Older Persons’ [7, 16, 17]. The Aged Care Clinical Mentoring (ACCM) defined as “a leader who facilitates improved quality of care for older people using best practice by providing and encouraging professional development in colleagues through communication, education and peer support” has been demonstrated an effective management practice model to promote the quality of care services [18], registered nurses served as clinical mentors for other nursing staff (mentees) and were responsible for leading evidence-based quality improvement programmes in four aged care facilities and four community aged care settings in two Australian states [19].. The ACCM is a specialized role that can encourage a mentee and provide them with available resources [20].. The ACCM model consists of six components that form five intervention strategies, including training geriatric care mentors, providing available training resources,promoting quality improvement process, establishing internal communication and feedback mechanism, providing expert support and external communication.(Fig. 1) The Aged Care Clinical Mentoring model (ACCM model) has been tested in four aged care facilities and four community aged care settings across two states in Australia [19, 21]. The results revealed that the ACCM model is an effective workforce model to improve aged care organizations’ capacity to create and sustain quality improvement [21].

Registered nurses are a minority in the workforce of nursing homes, with the majority of positions being held by nursing assistants [22]. However, most nursing assistants in Chinese nursing homes are not well educated, their social and economic status is usually lower than middle class, and most of them do not have knowledge of professional care concepts [23]. These are the factors that affect the implementation of evidence-based quality improvement in nursing homes. Wing to a paucity of ACCM models that have been developed for and tested in nursing homes, more evidence is needed on barriers to and enablers of a more effective implementation of the ACCM model in this environment.

Therefore, a Theoretical Domains Framework (TDF), which comprises 12 domains presenting determinants of behaviour, was applied to help translate our research results into clinical practice [24]. The TDF covers three different levels of individual, organizational and social factors, which is helpful for researchers to explore the influencing factors of behavior from different levels and predict the change of behaviour, and to understand the interviewees’ views and opinions on certain health behaviours, as well as the influencing factors in the process of promoting or hindering the implementation of the intervention [25, 26].. This study examines the perspectives of stakeholders of China’s nursing homes of the intervention prior to the ACCM model implementation, furthermore, enablers and barriers are summarized to provide a basis for the localization of ACCM model.

## Methods

### Design

An interpretive description approach [27] strengthened by thematic analysis was applied to the study to ensure that participants presented their perspectives on the mentoring model in more detail and to help the researchers interpret their perspectives. This method can also help researchers understand the relevant phenomena from the participants’ perspectives and experiences [28]. We adopted the 12 theoretical domains behaviour change framework for the improvement of evidence-based practice (Table 1) to develop interview questions and to capture the factors (barriers and enablers) that affect the implementation in a systematic way [24].

**Table 1:** Interview questions and the corresponding theoretical domains

Domain	Interview questions
Introductory questions	• Can you explain your understanding of care quality, evidence-based practice, and the mentoring model?
	• During your work, your overall impression of the care quality in this nursing home? (Prompts: current overall level, possible influencing factors, etc.)
knowledge	• To improve the care quality, what themes do you think should be given priority by the organization to nursing staffs? Or what aspects of training or mentoring would you most want to get?
skills	• What special skills do you need to promote care quality for the older people?
	• What are the difficulties in caring the older people and is there anything that would make it easier?
social/professional role and identity	• What is your current role in the organization?
beliefs about capabilities	• Do you think you can provide mentoring for mentees if you act as a mentor?
	• What factors reduce your confidence and what factors would help improve it?
beliefs about consequences	• Do you think the mentoring model is helpful in terms of the current care quality and training model in the nursing home? What might happen if the ACCM model were in place?
motivation and goals	• What is the main motivation for you to work and participate the training?
memory, attention and decision processes	• What factors will promote or hinder your active participation in the QI program? (Prompts: Or what factors do you think may affect your behaviour change after implementing the QI program)
environmental context and resources	• What factors in general practice do you consider make it easier or difficult to the implementation of QI program?
social influences	• Chinese nursing assistants have lower social status. Do you think nursing assistants will be interested in participating in the QI program?
	• What extent do you consider culture of practice in general practice facilitate or hinder the implementation of QI program in nursing home?
emotion regulation	• If the organization implements the ACCM model, how strongly do you feel about participating in the QI program?
behavioural regulation	• Does the nursing home currently have a systematic training model in place to facilitate behaviour change among nursing staffs to provide best care practices?
nature of the behaviour	• What systems do you think are needed in the general practice of promoting and sustaining behavior change for nursing homes?

### Participants

The study was conducted in Hunan Province and covered the four major prefecture-level cities of Changsha, Xiangtan, Zhuzhou, and Yueyang. Purposive sampling was used to select nursing staff who had rich caring experiences and nursing managers who had rich management experiences and to ensure a broad perspective from the nurse stakeholders [29].

The participants included in the study were clinical nurses with a minimum of 3 years of clinical work in a nursing home. We also included nursing managers who were registered nurses and who had more than 5 years of management experience. In addition, we included nursing assistants who possessed a qualification certificate and more than 3 months of work experience. The exclusion criteria were those who did not meet the above criteria or did not agree to participate in this study. The researchers made the announcement to the nursing homes firstly. The head of the nursing home who were interested in participating in this study notified the researchers by phone or text message. The administrators of large or very large nursing homes in Hunan province covering four major prefecture-level cities were randomly contacted, who agreed to participate in this study. Next, the researchers negotiated times and dates for interviews and focus group discussions with the participants. And then the researchers went to the nursing homes and asked the administrators to identify 6 eligible nurses and 12 eligible nursing assistants and invite them to a meeting where this study and the ACCM model of change were introduced. Consent was obtained prior to interviews and participants were free to withdraw from the study at any time. They were given an opportunity to ask any questions about this study. We did not stop contacting nursing homes for interviews until the novel themes became apparent [30, 31].

### Data collection

We conducted a pilot interview in February 2019 to test the interview questions, prompts, guides, and forms with a nursing home. Then, the researchers adjusted the interview guides according to the pilot study [16]; the detailed guides are attached in Table 1. Semi-structured interviews were carried out by two Masters of Nursing researchers (ZYN and LLL) between April and June 2019. One-to-one interviews were used with the nursing managers, and separate focus group discussions were used with the clinical nurses and nurse assistants. All of the interviews and focus groups were held in meeting rooms at the nursing homes where the participants were recruited. The interviews lasted for 20–30 min, and the focus group ranged from 40 to 60 min. All of the interviews were audio recorded and later transcribed verbatim. In addition, the first author documented the responses of every participant in the field notes during the interviews and focus groups.

### Data analysis

The data analysis and interpretation were guided by Braun and Clarke’s phases of thematic analysis [17]. First, the researchers coded the original data, and then they were categorized based on the framework of Michie et al. [24]. Any dilemmas that arose during the coding of the data were resolved through discussion and negotiation. The researchers identified themes and sub-themes during the research process. The basic characteristics of the participants are represented by usage rates or constituent ratios.

### Study rigor

The trustworthiness of the study was established by the following criteria: credibility, transferability, dependability, confirmability, and authenticity [32]. Credibility in the study was achieved by taping the interviews [33]. Four researchers and specialists in aged care formed an auditing panel in order to discuss the findings. The transferability of the study was improved by citing excerpts from the interviews to support the findings, looking for evidence to validate the findings, and delving deeper into the social context of Chinese nursing homes. To ensure the dependability of the analysis in the study, the transcripts were analysed by two researchers in the project, and the transcripts and thematic interpretations were also returned to the participants for verification [34]. The researchers established the confirmability of the study by linking explanations with the participants’ quotes. Finally, the verbatim transcripts and field notes were used to prove the authenticity of the study.

## Results

The researchers surveyed eight nursing homes. The participants included eight nurse managers, 50 clinical nurses and 64 nurse assistants. The participants’ demographic information is presented in Table 2. To guarantee the anonymity of the participants, every participant was tagged with a unique code. The eight nurse managers were identified as M 1–8, the 50 clinical nurses as N 1–50, and the 64 nursing assistants as A 1–64. Two major themes emerged to represent the views about the implementation of the ACCM model among participants in nursing homes. These themes were barriers to the implementation of the mentoring model and enablers of the implementation of the mentoring model. The main barriers include the sub-themes described as low educational background of the nursing assistants, the limitations of self-role orientation, resistance to change, lack of job motivation, and organizational constraints. The main enablers comprise the sub-themes of organizational support, the ability of nurses to apply evidence-based practice, proactivity, nursing supervision and feedback. These nine areas are discussed within the theoretical framework of Michie [24].

**Table 2:** The demographic information of participants (*n* = 122)

Participants’ characteristics	nurse manager (*N* = 8)	clinical nurse (*N* = 50)	nurse assistant (*N* = 64)	The total (N = 122)
Age ^†^	37.5 (35.0–55.2)	30.0 (26.7–32.2)	50.0 (43.0–52.0)	41.0 (30.5–50.5)
Gender ^‡^				
Male	2 (25.0)	0 (0.0)	1 (1.6)	3 (2.5)
Female	6 (75.0)	50 (100.0)	63 (98.4)	119 (97.5)
Qualification^‡^				
Elementary	0 (0.0)	0 (0.0)	12 (18.8)	12 (9.8)
Junior high	0 (0.0)	0 (0.0)	36 (56.3)	36 (29.5)
Senior high	0 (0.0)	0 (0.0)	14 (21.8)	14 (11.5)
Post-secondary	2 (25.0)	34 (68.0)	2 (3.1)	38 (31.2)
Undergraduate	6 (75.0)	16 (32.0)	0 (0.0)	22 (18.0)
Position^‡^				
Junior care assistant	0 (0.0)	0 (0.0)	36 (56.3)	36 (29.5)
Mediate care assistant	0 (0.0)	0 (0.0)	18 (28.1)	18 (14.7)
Senior care assistant	0 (0.0)	0 (0.0)	10 (15.6)	10 (8.2)
Nurse	0 (0.0)	20 (40.0)	0 (0.0)	20 (16.4)
Senior nurse	2 (25.0)	22 (44.0)	0 (0.0)	24 (19.7)
Supervisor nurse	6 (75.0)	8 (16.0)	0 (0.0)	14 (11.5)
Employment^‡^				
Temporary employment	0 (0.0)	14 (28.0)	0 (0.0)	14 (11.5)
Fixed-term contracts	4 (50.0)	34 (68.0)	64 (100.0)	102 (83.6)
Formal permanent	4 (50.0)	2 (4.0)	0 (0.0)	6 (4.9)
Years in the institution^†^	14.0 (12.2–33.7)	8.0 (5.0–10.2)	4.0 (2.0–6.0)	8.0 (3.0–10.0)
Daily working hours^†^	8.0 (8.0–8.0)	8.0 (8.0–8.0)	12.0 (10.0–12.0)	8.0 (8.0–12.0)

† median (IQR)

‡ n(%)

### Knowledge and skills

Some clinical nurses reported that the low educational background of the nursing assistants prevented them from absorbing knowledge quickly. This also results in their lower basic skill level and overall quality, poor learning and receptivity.

*“The educational background of many nursing assistants was low, even illiterate, so they didn't think over related factors like safety from the older people's perspective … You wanted him (nursing assistants) to write or read something, and he looked at the computer or books, and said oh no, I can't read it … ”* (N37)

Many nursing assistants reported that their expertise is limited and that they can only provide the most basic daily care for the elderly. According to the evaluation index of nursing quality in nursing institutions for the elderly, especially regarding the aspects of urinary incontinence, fall and stress injury, the nursing assistants do not have enough professional knowledge and skills to provide the best care for the elderly, and there are knowledge misunderstandings.

*“ … When we took care of those incontinent older people, we can only help him change diapers or the wet sheets. We didn't understand the pelvic floor exercises and the urination diary you mentioned … Incontinence in older people was normal, I thought we did not need to do some troublesome training or exercise because there was no cure!”* (A60)

*"Just roll over like that, put this pillow between his legs and his back... If there is a red area, massage it with safflower oil. If there is a broken skin, apply burn cream to it."* (A56)

*"... Then we have to tie him up. He can't walk steadily. When we won't let him go, he refuses to listen, he insists on walking, and when he does he always falls over. We can't be with him all the time, can we, when we have so many old people to look after?"* (A54)

### Social/professional role and identity (self-standards)

Several nurse managers had problems with self-role orientation, they believed that their seniority is higher than everyone else.

*“They (nursing assistants) had become accustomed to the original work pattern, and it was difficult for them to change it all at once … I tried to speak the truth with them, but they argued with me instead, and it ended up with me arguing with ten of them … They just thought they were older, and even though I was their leader, because I was new and young, they thought they could disobey me … ”* (M5)

*“They just think they are old, even though I am their leader, because I am new, I am young, they think they can disobey me... Then I think we need to understand that I am your leader and you have to listen to me and define your position."* (M5)

### Self-efficacy

The clinical nurses reported that when they encountered doubts at work, they would use evidence-based methods such as searching the Internet or books to overcome difficulties in work, which greatly stimulated their motivation to deal with difficulties and improved their ability and confidence.

*"... If I am in doubt about the accuracy of advice given, I will definitely look for evidence... I'm not just blindly doing everything."* (N19)

*“ … We will go surfing on the Internet or find related knowledge from books firstly (when in doubt at work), and then apply it to work practice … if the new way is more practical, we will stick with it … ”* (N20)

Nurses, especially senior nurses, believed that they were qualified to be mentors based on their capabilities. However, they also understand that their leadership skills need to be strengthened through, for example, training.

*“Our capabilities can still support us as their (nursing assistants) mentors in terms of professional knowledge … It's just that for me, I was still lacking in leadership ability and I didn't have a lot of leadership experience … ”* (N25)

*"Training is very important for us to further develop our capabilities... If the organization is willing to give regular training or send us out for further study, then we are very happy, haha..."* (N26)

### Anticipated outcomes/altitude

Some participants said resistance to behaviour change can make it difficult to implement programs effectively, especially in the initial stages. They were reluctant to attend professional training because they lack interest and have fixed thinking patterns. And some nursing assistants argued that there is no difference in practical outcomes between what nurses teach and what they currently implement, so they do not need to follow the nurse’s instructions.

*“ … Their (nursing assistants) training and change consciousness is very weak. They had their own manner of working. When I led them to care for the older people in a way which I thought was more reasonable, they didn’t agree with me, or they had other ideas … They always followed their own methods, which may hinder the effects of the training.”* (N23)

*"After all, we have taken care of the old man in our own way for so many years, and he seems to be very nice. It's a lot of trouble to change."* (A48)

### Intention

Almost all the nurses were supportive and excited about the description of the program.

*"If there is an opportunity (for quality improvement training intervention training program), we will definitely try to overcome the difficulties and actively participate in it. Quality of care has always been an important issue in the development of nursing institutions." (N49)*

*"It's the joy of learning. Well, it's also the desire to learn itself. We are all willing to learn from the experience of others. We have been in this organization all the time, and what we can see and learn is very limited. If we can absorb a lot of foreign experience, I think I can learn a lot." (N15)*

However, some nursing assistants reported that they treated this job as just a job and that they did not place considerable importance on their job. And other nursing assistants felt that they did not work to serve the older people or their own beliefs; rather, they only worked for salary.

*“ … if you asked him what he does for a living, and he would reply that he works as a hygienist, he would never reply that he was a nursing staff … they didn't have a self-identity, they felt like their job was just to help the older people pee and poop … ”* (N6)

*“ … I was so old, and I was retiring soon anyway, so I wasn’t willing to train, I was tired from my daily work … I worked, and I got paid my fair salary, but the training would take up too much of my extra time off … ”* (A37)

### Environmental constraints

Many participants reported that organizational support was helpful, particularly in the form of rewards for outstanding mentors or mentees and the implementation of regular meetings. All of the nurse managers showed that they were willing to try and support this QI program, which was helpful to care quality.

*“You might also build a reward machine, yeah, even if it was just a box of candy or chocolate, even open verbal praise at the meeting, but you can motivate them (mentees) to consciously implement care based on your guidance (best evidence-based practices) … They may just want to feel valued … ”* (N31)

*“ … If this ACCM model of change is helpful to improve care quality in the nursing home, then we still attach great importance to the training, but in view of the financial burden … well, you have to buy the equipment and materials, it was still a very heavy burden … But take the long view, it was useful for the development of the nursing home, and I was willing to support it … ”* (M4)

There was a consensus among the participants that organizational constraints such as lack of related resources, facilities, and time were main barriers. Some participants mentioned that time constraints created a barrier for them. Their limited time made it difficult for them to do their work and strictly follow the care procedure. Meanwhile, the lack of human resources was identified by participants. Many participants reported a remarkable shortage of nurses if each mentee was to be assigned a nurse mentor. The participants also discussed the limited organizational funding to support the project.

“*We (nursing assistants) were not enough, each of us had to care for about seven to eight older people … We were too busy, like a robot every day, so we had no time to train”* (A43).

*“The nurses in our organization were limited. There were only about four nurses working in each care unit every day … If each mentee was equipped with a nurse as a mentor, the number of mentors was certainly insufficient … ”* (M3)

*“The organizational funding was limited, but the market prices and human costs were high. It is true that our organization lacks such professional mentors, but it is unrealistic for us to recruit more authorized nurses as mentors, and we didn't have much money to bring in many experts … ”* (M2)

Some clinical nurses mentioned that only implementing short-term intensive training made it difficult for them to provide more professional knowledge to nursing assistants. In addition, they said that the nursing assistants were too busy to regularly attend trainings.

*“ … The turnover rate of nursing assistants was high, so we spent most of our time training on normal operations, especially when they were new to the job … But we just taught them how to do it for a while, and then they quit, so … ”* (N24)

### Emotion

Some nursing assistants reported that they were highly aware of the importance of knowing care principles that could be learned from senior nursing assistants and nurses.

*“ … We hoped to have more opportunities to communicate with you and exchange information about our care experience with each other … I wanted to learn from them how to take care of the older people more easily. Ah, we learned these skills, which can be better applied to our work.”* (A6)

### Behavioral regular

Some participants discussed the value of nursing supervision and the feedback. Close teamwork between nursing assistants and nurses is beneficial to the implementation of ACCM model.

*“At present, our facility had implemented a regular examination assessment, which was not to evaluate whether you can answer the question on an examination paper … Your test answers were correct, but your actual operation at work remained the same as before, without any improvement … That meant we had to … Ah, we had to check, and then be periodically evaluated in our daily work.”* (N40)

Some nursing assistants suggested that assigning the most experienced and enlightened nursing assistants at each care unit as site champions, who would monitor other nursing assistants (mentees) and share feedback with the mentors.

*“ … They (nurse managers) were so busy that we didn't always meet with them. But if one of us (nursing assistants) was assigned as, ah, a site champion, I thought we could communicate better … which can be conducive to maintaining the effectiveness of training.”* (A15)

## Discussion

The 12 theoretical domains for improving evidence-based practice provided a conducive perspective through which to view enablers and barriers to the effective implementation of the QI program based on ACCM model in nursing homes. The biggest enablers were the proactivity of the participants to the project and managerial support. The biggest barriers were the time constraints of the nursing assistants and the lack of funding. These findings help address gaps in research and reality, and lay a foundation for further promotion and development of the ACCM model of change in nursing homes [35].

The findings provide fresh evidence that participants’ proactivity to engage in a project is related to their opportunity to gain more organizational support and support from clinical mentors [36]. However, the fixed mind set of the nursing assistants, which leads them to resist training and behaviour change, is a major obstacle. Studies have confirmed the importance of motivation to behaviour change and shown that the mentee’s and mentor’s degrees of proactivity are of primary importance to the establishment of positive mentoring relationships [37]. [38] The head of an organization can set up a reward mechanism to help improve the proactivity of the participants [39]. Administrative support is an additional enabler of implementing the mentoring model, especially in reaching sustainable development and maintenance. It has been suggested that in order to gain their full support, the organization administrators can serve as members of the project steering committee [20].

Most participants believed that the learning environment and resources were crucial to the QI program. In recognition of the fact that the facilities of some nursing homes are poor, the development of an online training was considered an effective and flexible way to alleviate the barrier of access to the specialized progress (i.e., cost, convenient time and training site) [40, 41]. Meanwhile, we can promote participation in various styles of training (combined online and offline training). For example, we can implement a routine training, which focuses on 10–15 min of education during the morning meeting. We can also continue the practice guidance at work, which underlines the compatibility between theory and practice, as both are the basis of critical systems thinking [42]. In addition, unlike in Australia, most Chinese nursing assistants are older and have a low educational background, [43] therefore, we can use a more simplified manual that combines pictures and texts could be used to help implement the education for the mentee, which should also be evidence-informed and readable [44].

Self-role orientation disorder for nursing assistants was identified as a hindrance to effective mentorship, which can be eased by building matched expectations and long-term communication between mentors and mentees. Study showed that the internal process of the mentorship system is the distribution of power among mentoring relationships. Mentoring relationships seem to be associated with issues of organizational justice and power, as they were assumed to sometimes conceal poor functioning of the mentee and give mentee access to information in the organization which is not available for other coworkers [45, 46]. Our research discovered willingness among clinical nurses and nursing assistants, especially senior clinical nurses, to undertake a different role in the clinical field [47]. Managers should also identify and expand the roles and responsibility of each mentee and mentor and empower them accordingly [48].

Nursing supervision and feedback were also considered as enablers. In Australia, research has shown that the identification of mentors in every nursing home that would receive sustained education and a refresher course training would be helpful [49]. Due to the shortage of nurses, senior nursing assistants in each care unit could be appointed as site champions to supervise other nursing assistants and provide regular feedback [47]. Nursing supervision and feedback could provide nursing staff with opportunities to learn from others [50].

The research strived to present a suitable evidence-based practice model in nursing homes in China. We discovered how mentors and mentees can collaborate to promote the best evidence-based practice and that professional educational teaching materials and necessary financial support are needed for successful implementation [51]. The development of information technology makes it possible to consider combining online training with field training to facilitate continuous care [50].

This study had some limitations. The sample was almost all women with an average age of 41. Because in China, the women is more family-oriented [52], this may affect the motivation for participation, particularly in relation to role management.

As far as we know, the study is the first study dedicated to identifying enablers and barriers to implement QI program based on ACCM model of change in the Chinese nursing home. Our findings indicate that nursing staff’s motivation, organizational support and environment are crucial to the successful implementation of the model. To make a change from the existing training model demands corporate efforts at both the organizational and individual levels. The form of the mentoring can be flexible based on the needs of nursing assistants or new nurses.

## Data Availability

The datasets used or analyzed during the current study are available from the corresponding author on reasonable request.

## Abbreviations

ACCM: aged care clinical mentoring
QI: quality improvements
