# Correction to: Enablers and barriers to implementing care quality improvement program in nursing homes in China

**DOI:** 10.1186/s12877-021-02658-0

**Published:** 2022-01-03

**Authors:** Yinan Zhao, Lulu Liao, Hui Feng, Huijing Chen, Hongting Ning

**Affiliations:** 1 Xiangya School of Nursing, Central South University, Changsha, Hunan China; 2 Xiangya-Oceanwide Health Management Research Institute, Central South University, Changsha, China; 3 National Clinical Research Center for Geriatric Disorders, Xiangya Hospital, Changsha, China

**Correction to: BMC Geriatr 21, 532 (2021)**

**https://doi.org/10.1186/s12877-021-02488-0**

During the publication process of the original article [1] the following contribution statement was accidentally omitted:

– Yinan Zhao and Lulu Liao equally contributed to this work.

The publisher apologizes for the inconvenience caused. The original article [1] has been updated.
